# Equilibrium and Kinetics of Sin Nombre Hantavirus Binding at DAF/CD55 Functionalized Bead Surfaces 

**DOI:** 10.3390/v6031091

**Published:** 2014-03-10

**Authors:** Tione Buranda, Scarlett Swanson, Virginie Bondu, Leah Schaefer, James Maclean, Zhenzhen Mo, Keith Wycoff, Archana Belle, Brian Hjelle

**Affiliations:** 1 Department of Pathology, University of New Mexico School of Medicine, MSC08 4640, Albuquerque, NM 87131, USA; E-Mails: scswanson@salud.unm.edu (S.S.); vbondu@salud.unm.edu (V.B.); bhjelle@salud.unm.edu (B.H.); 2 Cancer Center, University of New Mexico School of Medicine, MSC08 4640, Albuquerque, NM 87131, USA; 3 Center for Infectious Diseases and Immunity, University of New Mexico School of Medicine, Albuquerque, NM 87131, USA; 4 Planet Biotechnology Inc., 25571 Clawiter Road, Hayward, CA 94545, USA; E-Mails: leah.Schaefer@gmail.com or lschaefer@planetbiotechnology.com (L.S.); jmaclean@planetbiotechnology.com (J.M.); zmo@planetbiotechnology.com (Z.M.); kwycoff@planetbiotechnology.com (K.W.); abelle@planetbiotechnology.com (A.B.)

**Keywords:** DAF/CD55, hantavirus, co-receptor, kinetics, equilibrium binding, flow cytometry

## Abstract

Decay accelerating factor (DAF/CD55) is targeted by many pathogens for cell entry. It has been implicated as a co-receptor for hantaviruses. To examine the binding of hantaviruses to DAF, we describe the use of Protein G beads for binding human IgG Fc domain-functionalized DAF ((DAF)_2_-Fc). When mixed with Protein G beads the resulting DAF beads can be used as a generalizable platform for measuring kinetic and equilibrium binding constants of DAF binding targets. The hantavirus interaction has high affinity (24–30 nM; k_on_ ~ 10^5^ M^−1^s^−1^, k_off_ ~ 0.0045 s^−1^). The bivalent (DAF)_2_-Fc/SNV data agree with hantavirus binding to DAF expressed on Tanoue B cells (*K_d_* = 14.0 nM). Monovalent affinity interaction between SNV and recombinant DAF of 58.0 nM is determined from competition binding. This study serves a dual purpose of presenting a convenient and quantitative approach of measuring binding affinities between DAF and the many cognate viral and bacterial ligands and providing new data on the binding constant of DAF and Sin Nombre hantavirus. Knowledge of the equilibrium binding constant allows for the determination of the relative fractions of bound and free virus particles in cell entry assays. This is important for drug discovery assays for cell entry inhibitors.

## 1. Introduction

Virus entry and productive infection generally require the expression of specific cell-surface receptor molecules on target cells. Receptors can efficiently target viruses for endocytosis or may be used to activate specific signaling pathways that facilitate entry. Decay-accelerating factor (DAF/CD55) has been implicated in many cases of multi-receptor tropism amongst the enteroviruses [1,2,3,4,5,6,7,8,9,10,11,12,13], and other microbial proteins [14]*.* Thus, extensive structural and biochemical studies of DAF interactions with various serotypes of Enteroviruses (EV) and Group B Coxsackieviruses (CVB) have presented mechanistic insights into how DAF functions as a co-receptor for enteroviruses [8,9,10,11,12]. 

More recently, DAF has been identified as co-receptor of pathogenic hantaviruses: Hantaan virus (HTNV), Puumala virus (PUUV) [15,16] and Sin Nombre virus (SNV) [17]. α_V_β_3_ integrin is generally known as the primary endocytic receptor for pathogenic hantaviruses which include: HTNV, Seoul virus (SEOV), PUUV, SNV, and New York-1 virus (NYV) [18]. Pathogenic hantaviruses cause hemorrhagic fever with renal syndrome (HFRS) and hantavirus cardiopulmonary syndrome (HCPS), with case fatality rates for HCPS generally ranging from 30%–50%. This study is primarily focused on SNV, which was first isolated in the Southwestern region of the U.S. and carried by the deer mouse *Peromyscus maniculatus*. It is the primary causative agent of HCPS in North America [19,20,21,22]. 

**Figure 1:**
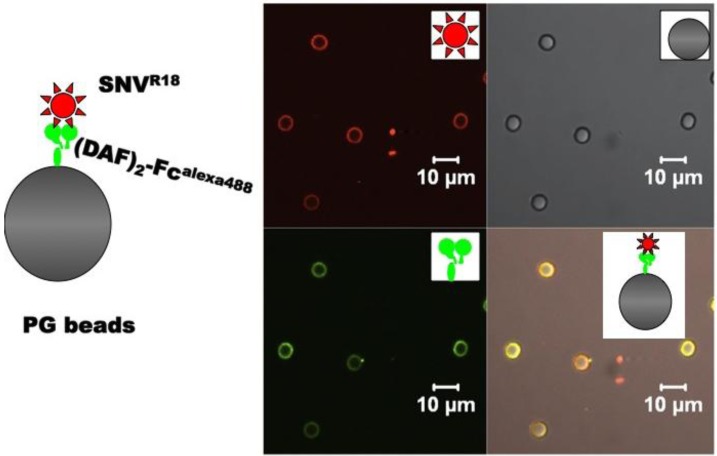
Schematic of the molecular assembly of Alexa 488 labeled, IgG Fc domain-functionalized DAF (DAF)_2_-Fc^Alexa488^ and octadecyl rhodamine B (R18)-labeled SNV (SNV^R18^) on Protein G beads and confocal microscopy images of the components. (DAF)_2_-Fc is immobilized on protein G (*K_d_* = 12 nM) and then used to capture and display fluorescently labeled UV killed SNV.

To the best of our knowledge the interactions of hantaviruses and DAF have been limited to few functional (infection) assays in the literature [15,16,17,23]. In this study we measure equilibrium and kinetic binding constants of killed SNV to purified IgG Fc domain-functionalized DAF ((DAF)_2_-Fc) proteins immobilized on protein G beads. This paper serves a dual purpose of presenting a convenient and quantitative approach of measuring binding affinities between DAF and the many cognate viral and bacterial ligands, and providing new data on the binding constant of DAF and Sin Nombre hantavirus (Figure 1). Knowledge of the equilibrium binding constant allows for the determination of the relative fractions of bound and free virus particles in cell entry assays [24]. This is important for drug discovery assays for cell entry inhibitors. *In vivo*, ligand-receptor interactions are mostly governed by non-equilibrium conditions [25] unless the characteristic time to equilibrium is fast, <1 s [26,27]. In this way kinetic measurements can be used to gain mechanistic insights into how viruses interact with cognate receptors, and make it possible to make useful comparisons with other receptor-ligand interactions. The equilibrium and kinetic measurements of killed SNV with DAF on beads are shown to provide a reasonable model for understanding and inhibiting productive infection. 

## 2. Results and Discussion

### 2.1. Molecular Assembly of (DAF)_2_-Fc^Alexa488^ on Beads: Equilibrium and Kinetic Parameters

**Figure 2:**
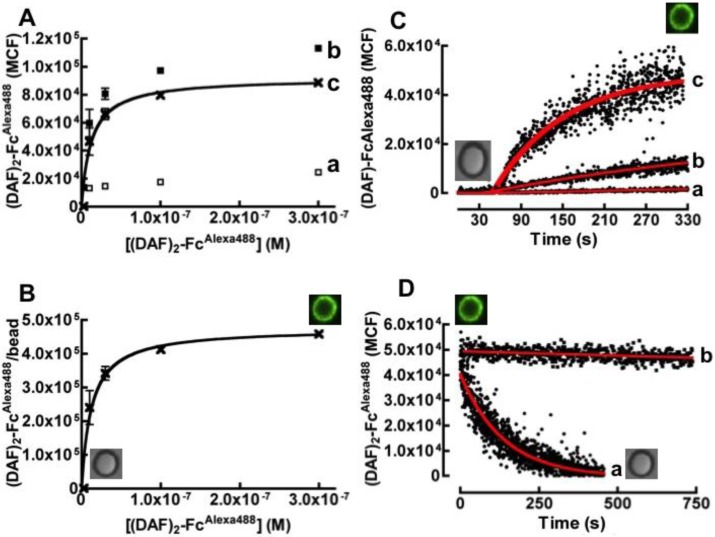
Equilibrium binding analysis of (DAF)_2_-Fc^Alexa488^ to protein G beads. (**A**) Plot of bound (DAF)_2_-Fc^Alexa488^* versus* concentration of soluble (DAF)_2_-Fc^Alexa488^. (**a**) Non-specific binding of various titers of (DAF)_2_-Fc^Alexa488^ were mixed with 10,000 streptavidin coated beads in 20 µL, (**b**) Total binding and (**c**) Specific binding of (DAF)_2_-Fc^Alexa488^ to 10,000 protein G beads. (**B**) Hyperbolic plot of (DAF)_2_-Fc^Alexa488^ molecules/protein G bead *versus* concentration of soluble (DAF)_2_-Fc^Alexa488^. The site occupancies were determined using Mean Equivalent of Soluble Fluorophores (MESF) standard calibration beads as described in the Experimental Section. The data were fit to simple Langmuirian binding curve to yield a *K_d_* of 12 nM. (**C**) Kinetic analysis of binding of: (**a**) 2.43 × 10^−1^° M, (**b**) 2.43 × 10^−9^ M, and (**c**) 2.43 × 10^−8^ M of fluorescently labeled (DAF)_2_-Fc to 40,000 beads in 400 µL by flow cytometry. The increase in bead-associated fluorescence over time was analyzed by the kinetic method of initial rates [30] to yield the following rate constants: (**a**) 6.90 × 10^5^ M^−1^s^−1^ (**b**) 5.16 × 10^5^ M^−1^s^−1^ (**c**) 6.71 × 10^5^ M^−1^s^−1^. (**D**) Dissociations of (DAF)_2_-Fc^Alexa488^ from beads. (**a**) Dissociation kinetics induced by competition with a large excess of soluble Protein G added to molecular assembly. The data were fit to a single exponential decay curve to yield *k_off_* = 0.007 s^−1^. (**b**) The molecular assembly is relatively stable in the absence of a competitor. The square inserts are photographs of non-fluorescent and fluorescent beads or cells under different experimental conditions.

Binding of fluorescently labeled (DAF)_2_-Fc^Alexa488^ to protein G beads was measured by incubating a variety of concentrations of the fluorescent probe with fixed aliquots of beads and analyzing the samples on a flow cytometer. Figure 2A shows hyperbolic plots of median channel fluorescence (MCF) of bead bead-borne (DAF)_2_-Fc^Alexa488^* versus* initial concentration of (DAF)_2_-Fc^Alexa488^. The three curves represent non-specific binding to streptavidin-coated beads (a in Figure 2A), and total binding to Protein G beads (b in Figure 2A) and specific binding to Protein G beads (c in Figure 2A). Specific binding was calculated as the difference between total and non-specific binding curves. The data show that non‑specific binding to naked streptavidin-coated protein G beads was minimal over the concentration range of our experiments. Figure 2B shows a hyperbolic plot of various (DAF)_2_-Fc^Alexa488^/bead site occupancies *versus* their initial concentration of (DAF)_2_-Fc^Alexa488^. Analysis of the binding curve yielded an affinity constant of 12.0 nM. The maximum effective site occupancy of (DAF)_2_-Fc^Alexa488^ was determined to be ~225,000 sites/bead.

Figure 2C shows an overlay of bead binding time course of different concentrations of (DAF)_2_-Fc^Alexa488^ to 40,000 beads in 400 µL samples. We used the site-occupancy data to establish a simple bimolecular kinetic model, describing the interaction between protein G sites and the Fc domain of (DAF)_2_-Fc^Alexa488^ to fit the data and solve for the binding rate constant (*k_on_*). The analysis yielded an average binding rate constant of *k_on_* = (6.2 ± 0.8) × 10^5^ M^−1^ s^−1^, where the error is the standard deviation of three separate measurements. Figure 2D shows single exponential fit to a dissociation curve generated by a large excess (1000 × *K_d_*) of soluble Protein G added to the molecular assembly; *k_off_* = (7.0 ± 0.3) × 10^−3^ s^−1^. In the absence of a competitor (curve b in Figure 2D), this molecular assembly was robust due to facile rebinding of the ligand [28] and remained wholly stable for days, allowing for the long-term storage of Protein G/DAF bead stocks. This affinity constant derived from kinetic data (*K_d_* = *k*_off_/*k*_on_ = 11.3 nM) was compatible with the equilibrium binding result. Collectively, these binding parameters are comparable to kinetic and equilibrium constants of Protein G interactions with antibody Fc domains [29].

In summary, we have developed a generalizable molecular assembly platform for studying interactions between DAF and its ligands (*cf.* Figure 1). Because DAF is a molecular target of many viral and bacterial pathogens, the approach described herein, has the potential to be applicable to a wide range of systems. The bead platform involves non-covalent immobilization of (DAF)_2_-Fc on beads in a relatively simple process governed by mass action, e.g., simple mixing of Protein G beads with (DAF)_2_-Fc reaches equilibrium within minutes (Equation (1)) [27]:

(*t_eq_* = 3.*5*/(*k_on_*[(DAF)_2_-Fc] *+ k_off_*))**(1)

As an illustrative example, the equilibration times (calculated from Equation (1)) for incubating (DAF)_2_-Fc with Protein G beads using the same concentration of soluble (DAF)_2_-Fc, as that used in Figure 2C are: (a) ~8 min; (b) ~7 min; (c) ~3 min.

### 2.2. Binding between DAF and SNV on Beads and Tanoue B Cells Is Governed by Comparable Equilibrium Dissociation Constants

**Figure 3:**
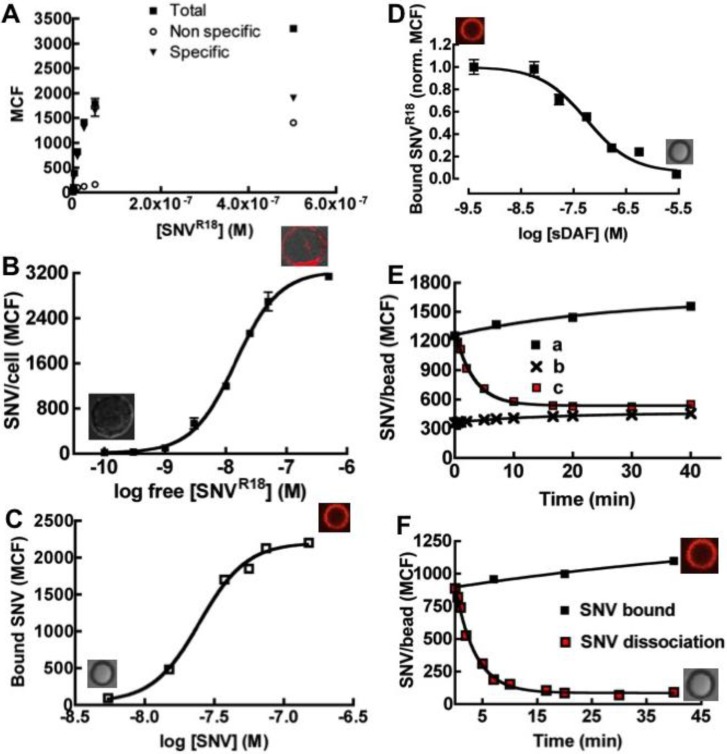
Binding of SNV^R18^ to Tanoue B cells and DAF beads. (**A**) Equilibrium binding of SNV^R18 ^to 10,000 Tanoue B cells in 10 µL. A plot of median channel fluorescence (MCF) of cells incubated with SNV^R18 ^(Non-specific, Total and Specific) *versus* the concentrations of SNV^R18^ added. (**B**) The specific data from (**A**) recast with log free [SNV^R18^] on the x-axis. This gives a sigmoid curve, with *K_d_* = 14.0 nM. (**C**) Equilibrium specific binding of SNV^R18^ to DAF beads yielding a *K_d_* ~ 24 nM. (**D**) Competition binding curve using a fixed quantity of SNV^R18^ and various concentrations of soluble DAF (sDAF), giving a *K_i_* = 58.0 nM. (**E**) Dissociation of bound SNV^R18^ from DAF beads, induced by 10-fold excess of unlabeled SNV. (**a**) Total binding of SNV^R18^ to (DAF)_2_-Fc beads, no additions. (**b**) Non-specific binding to Protein G beads. (**c**) Addition of 10-fold excess of SNV to SNV^R18^-bearing DAF beads induces dissociation of SNV^R18^. (**F**) Baseline corrected dissociation of SNV^R18^ from DAF beads. The data was fit to a single exponential decay, yielding *k_off_* = 0.0045 s^−1^.

Tanoue B-suspension cells express DAF but not the entry receptor integrin α_v_β_3 _(Table 1). These cells are suitable substrates for studying sole DAF/SNV interactions separately from α_v_β_3_. We therefore examined the equilibrium binding characteristics of SNV^R18^ to DAF-bearing beads in comparison to DAF-expressing Tanoue B cells. While we have previously analyzed the binding of SNV^R18^ to Tanoue B cells, we recapitulate those measurements herein because the earlier study reported an anomalously low dissociation constant, *K_d_* of 26 pM [17] for this interaction, because that study failed to accurately assess the valency of free virus particles (562 Gn-Gc heterodimers on SNV, see Methods). The binding of SNV^R18^ titers was plotted as a hyperbolic graph in Figure 3A. The data were corrected for non-specific binding and were then replotted as log-transformed sigmoidal dose response-curves of median channel fluorescence intensity of bound SNV^R18^* versus* the concentration of SNV^R18^ particles in solution (Figure 3B). The result of a fit, *K_d_* = 14.6 nM, was closely correlated to the (DAF)_2_-Fc on beads which yielded an effective affinity constant of 24.7 nM (Figure 3C). This suggested that the mode of SNV^R18^ binding to cell-expressed DAF and (DAF)_2_-Fc beads was comparable. The monovalent affinity of sDAF to SNV^R18^ was determined from competitive binding experiments between soluble recombinant DAF (sDAF) and (DAF)_2_-Fc. The inhibitor constant, *K_i_*, was determined from the competition binding curve (Figure 3D) using the equation of Cheng and Prusoff [31] embedded in Graphpad Prism software [32]. The analysis yielded a *K_i_* value of 58.0 nM. We next measured the dissociation rate of SNV from DAF on beads by using unlabeled SNV as a competitor. Fluorescently tagged SNV^R18^ immobilized on (DAF)_2_-Fc beads was competed off the beads with a ten-fold excess of unlabeled SNV which was added to the sample. Intensity readings were taken at various time intervals for over 40 minutes. The loss of intensity (c in Figure 3E) was plotted together with the total binding (a in Figure 3E) and non-specific binding (b in Figure 3E) over the time course. The dissociation curve was fit to a single exponential decay, which yielded a dissociation rate constant of 0.27 min^−1^ or 0.0045 s^−1^.

**Table 1:** Total Receptor surface distribution of DAF and α_v_β_3 _at Vero-E6, and Tanoue B cells surfaces.

Cell	DAF ^a^	Total α_v_β_3 _^b^
Vero E6	5.85 ± 1.37 × 10^3 i^	1.61 ± 0.38 × 10^6^
	2.54 ± 0.84 × 10^5 ii^	
	2.26 ± 0.28 × 10^5 iii^	
Tanoue B	9.29 ± 2.18 × 10^4 i^	None detected

Median channel fluorescence (MCF) from flow cytometry histograms was used to determine the receptor expression using MESF calibration beads (see Experimental methods). The same secondary antibody was used for all measurements. IgG1 and IgG 2a K isotype controls were used as appropriate isotype controls. (**a**) Several primary antibodies were used because of concerns about poor cross-species reactivity between the commercially available anti anti-human DAF clone BRIC 216 and DAF expressed on green monkey (Vero E6) cells. The results from the different clones are listed: (i) clone BRIC 216 (Millipore) (ii) IA10 (iii) 2H6 ascites. (**b**) Anti-Integrin α_v_β_3_ antibody, clone 23C6, was used as primary antibody. Secondary antibody was an Alexa488 tagged goat anti anti-mouse. Adherent cells (Vero E6) were detached with Accutase (Sigma, St. Louis, MO, USA), blocked with 10% human serum for 1 h, and incubated with primary antibodies at 4 °C on a rotator for 2 h. Secondary antibodies were incubated at 4 °C on a rotator for 1 h. The cells were washed once and then analyzed with an Accuri C6 flow cytometer. Non-specific binding by secondary antibodies was determined by staining cells in the absence of primary antibodies.

#### Kinetic Binding Analysis

**Figure 4:**
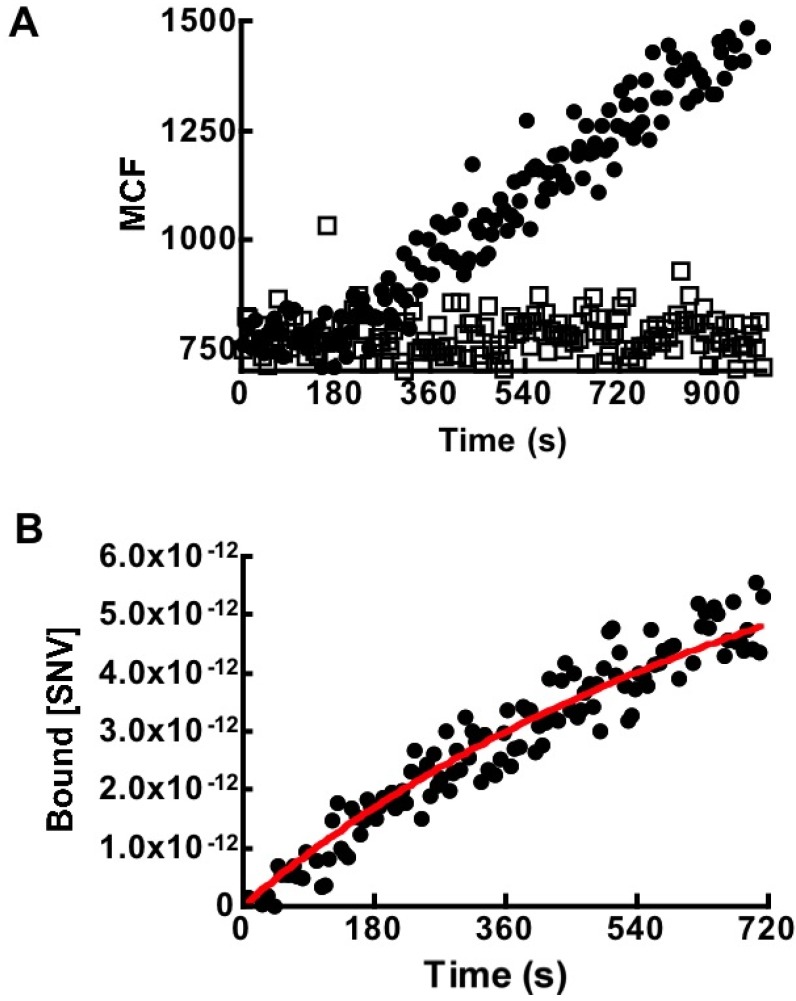
(**A**) Kinetic analysis of binding of 4 pM virus to 40,000 Protein G beads ± (DAF)_2_-Fc in 400 µL by flow cytometry. Association with naked protein G beads shows negligible non-specific binding between viral particles and beads. (**B**) Kinetic modeling (Equation (5)) of SNV^R18^ binding data. The average results and standard deviation for three different experimental curves were *k_on_* = (6.05 ± 0.45) × 10^4^ M^−1^s^−1^ when *k_off_’* was fixed at 0.0045 s^−1^, as derived from Figure 5F.

Real time binding time courses of 10, 20, and 30 µL aliquots of 10^8^/µL SNV^R18^ particles to 40,000 (DAF)_2_-Fc beads in 400 µL HHB buffer were analyzed on a flow cytometer. Figure 4A shows an overlay of 30 × 10^8^ SNV^R18^ particles binding to (DAF)_2_-Fc beads (a in Figure 4A) and Protein G beads (b in Figure 4A). The non-specific binding of SNV^R18^ to Protein G beads was subtracted from the total binding to (DAF)_2_-Fc beads. 

The typical data were averaged over 10-second time bins to reduce the noise and then fit to the model given in Equations (4) and (5). The median channel fluorescence (MCF) associated with SNV^R18^ was then converted to surface occupancy of SNV^R18^/bead using lipobead calibration standards as described in the Experimental section. The data were then converted to concentration units as previously described [27]. The dissociation rate constant for SNV^R18^/DAF, which we determined from experiment (*k_off_* = 0.0045 s^−1^; Figure 3F) was used as a fixed parameter in the kinetic model; Equation (5). This allowed the simplification of the kinetic analysis of the kinetic data to a single parameter (*k_on_*) fit employing least-squares minimization between Equation (5) and experimental data. The result yielded an average binding rate constant of *k_on_* = (1.5 ± 0.5) × 10^5^ M^−1^s^−1^, where the errors are the standard deviations for three experimental runs using different [SNV^R18^]_0_. Thus, the *K_d_* = 30.0 nM derived from the ratio of k_off_/k_on_—(*i.e.*, microscopic reversibility) [30] closely agrees with the 24.7 nM value which we derived from equilibrium binding measurements (Figure 3C). The results from this study suggest that SNV binds to DAF with higher affinity compared to micromolar range affinities that have been determined for some DAF binding enteroviruses [6,33,34]. 

### 2.3. Binding Parameters Derived from Bead Platform Provide a Reasonable Model for Infection Inhibition

Equilibrium binding constants of SNV to DAF allow quantitative estimates of inhibitor concentrations to be made according to Equation (2) [35]:

*θ =* ([*DAF*]*_free_*/*K_d_*)/(*1+*([*DAF*]*_free_*/*K_d_*)) (2)

**Figure 5:**
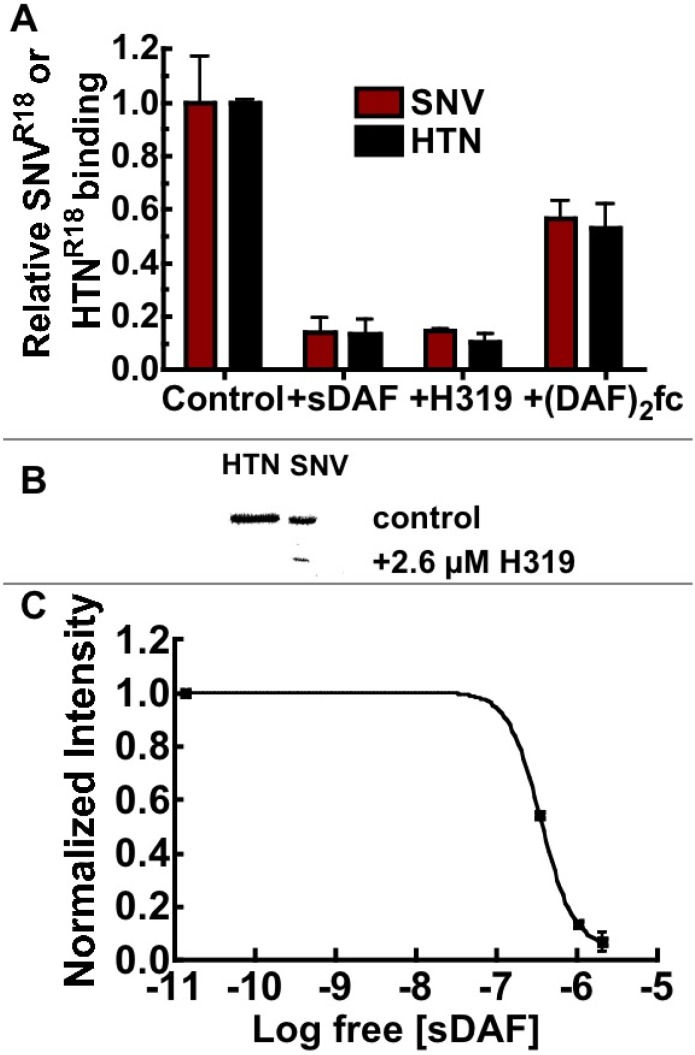
(**A**) Soluble DAF (sDAF) is significantly better than (DAF)_2_-Fc at blocking cell binding and entry of SNV. Flow cytometry analysis of Tanoue B cell-binding inhibition of SNV^R18^ or HTN^R18^ using 1.0 µM sDAF, 26 µM H319 polyclonal antibody against DAF and 18.0 µM (DAF)_2_-Fc. sDAF and H319 inhibited cellular binding of SNV and HTN by >90% compared to <50% associated with (DAF)_2_-Fc. (**B**) Western blotting of Vero E6 cells infected with HTN and SNV (control) and cells pretreated with 2.6 µM H319 anti-DAF antibody. (**C**) Analysis of inhibition of infection of Vero E6 cells by SNV with sDAF, measuring viral N-protein by western blot, which is presented as of a plot of normalized intensity *versus* concentration of sDAF. Quantitative analysis of the gel was performed using a Biorad Molecular Imager, ChemiDoc XRS+ equipped with *Image Lab Software 4.1* [38].

In this way, we assessed the ability of sDAF and (DAF)_2_-Fc to block the binding of hantavirus to Tanoue B cells expressing 100,000 DAF/cell, and lacking α_v_β_3_ expression (Table 1). As noted in the introduction, the first description of molecular recognition of DAF by hantaviruses was associated with Hantaan (HTN) and Puumala [15]. UV killed fluorescently labeled HTN and SNV were tested in parallel in binding inhibition experiments using large excesses of sDAF (~1 µM), (DAF)_2_-Fc (18 µM), and 26 µM H19 anti DAF polyclonal antibody. Paired samples of 3.0 × 10^7^ fluorescently labeled virusparticles were used in each case. The concentration of Gn-Gc heterodimers on the virus particles [36,37] was estimated to be 1.4 nM in 20 µL (see methods; Figure 7). The pre-blocked SNV^R18^ samples were then incubated with 10,000 Tanoue B cells in 20 µL for 30 minutes before the cells were analyzed with a flow cytometer. The results showed that sDAF, used in excess, inhibited SNV^R18^ binding by >90% compared to ~50% for (DAF)_2_-Fc (Figure 5A). The results show that excess sDAF efficiently blocks binding of virus particles when in competition with the lower concentrations of DAF expressed on Tanoue B cells (1.67 × 10^−15^ moles (83.5 pM)). However, the failure of two-footed (DAF)_2_-Fc to efficiently block binding to cells might be attributable to orientation constraints [34] relative to the tetrameric presentation of Gn and Gc structures on virus surfaces [36,37]. This idea is supported by the sDAF-comparable capacity to inhibit cell binding as monomers that were derived from papain-digestion of (DAF)_2_-Fc. We then compared the ability of H319 to block infection of Vero E6 by HTN and SNV. As shown for HTN and other Old World hantaviruses, elsewhere [15,16], H319 blocked infection of cells by HTN and SNV (Figure 5B). 

Finally, we used increasing titers of up to 3.35 µM soluble DAF (sDAF) to inhibit infection of Vero E6. It is worth noting that the ratio of total particles to infectious virions in our preparations is 14,000:1 [17]. As infectious and non-infectious particles are equally capable of receptor occupancy, a multiplicity of infection (moi) of 0.1 applied to 150,000 cells is equivalent to 2.1 × 10^8^ particles or 19.6 × 10^−9^ M Gn-Gc receptors in 100 µL. Thus, 3.35 µM sDAF is theoretically high enough to inhibit SNV from binding to cellular DAF and α_v_β_3_ (Table 1) with which it is in competition for binding to SNV glycoproteins. In this way the sDAF inhibited infection by ≥80% at the highest concentration (Figure 5C). The infection results are comparable to the blocking of infection by old world hantaviruses with sDAF and H319 anti DAF antibodies [15,16]. In conclusion this study has established a robust platform that has the potential of measuring binding interactions of DAF and many of its ligands as noted in the introduction. 

## 3. Experimental Section

### 3.1. Materials

Octadecyl rhodamine B chloride (R18), and 5-octadecanoylaminofluorescein (F18) were purchased from Molecular Probes (Life Technologies, Eugene, OR, USA) and used without further purification. 6.8 µm Protein G coated polystyrene beads were purchased from Spherotech (Libertyville, IL, USA). Recombinant DAF was purchased from R&D systems. Rabbit polyclonal H319 anti DAF antibody was purchased from Santa Cruz Biotechnology (Dallas, TX, USA). Primary antibodies for immunostaining: CD55/DAF (1:40, Millipore Cd55 MsxHu, # CBL 511) mouse monoclonal anti‑human DAF, clones IA10, 2H6 ascites, 8A7 ascites [12], mouse monoclonal anti-integrin α_v_β_3_ (1:40, Millipore MsxHu, # MAB 1976), Secondary antibodies: (anti-mouse IgG Alexa488, anti-mouse IgG Alexa647, anti-mouse IgG Cy5, anti-rabbit Alexa647; all from Life Science Technologies, (Carlsbad, CA, USA). Amine-reactive Alexa Fluor 488 carboxylic acid, succinimidyl ester probe was purchased from Life Technologies (Carlsbad, CA, USA). Phosphate-buffered saline (PBS) was purchased from Mediatech, Inc, (Herndon, VA, USA). Dimethyl sulfoxide (DMSO) and Sephadex G-50 were purchased from Sigma. TRIS (10 mM or 25 mM Tris, 150 mM NaCl, pH 7.5) and HHB (30 mM HEPES, 110 mM NaCl, 10 mM KCl, 1 mM MgCl_2_6H_2_O and 10 mM glucose, pH 7.4) buffer, and Hanks Balanced Saline Solution (HBSS) (0.35 g NaH_2_CO_3_, 0.049 g MgSO_4_, 1 mM CaCl_2_ or 1 mM MnCl_2_) were prepared under sterile conditions and stored in 50 mL tubes at −20 °C. 

### 3.2. Production of Sin Nombre Virus

SNV was propagated and titered in Vero E6 cells under strict standard operating procedures using biosafety level 3 (BSL3) facilities and practices (CDC registration number C20041018-0267) as previously described [39]. For preparation of UV-inactivated SNV, we placed 100 µL of virus stock (isolate SN77734, typically 1.5–2 × 10^6^ focus forming units/mL) in each well of a 96-well plate and subjected the virus to UV irradiation at 254 nm for various time intervals (~5 mW/cm^2^) as described elsewhere [17]. We verified efficiency of virus inactivation by focus assay before removing from the BSL-3 facility.

### 3.3. Fluorescent Labeling of SNV

The envelope membrane of hantavirus particles was stained with the lipophilic lipid probe octadecylrhodamine (R18) and purified as previously described [17]. The typical yield of viral preparation was 1 ± 0.5 × 10^8^ particles/µL in 300 µL tagged with ~10,000 R18 probes/particle or 2.7 mole% R18 probes in the envelope membrane of each particle of 192 nm diameter average size [17]. Samples were aliquoted and stored in 0.1% HSA HHB buffer, and used within two days of preparation and storage at 4 °C. For long-term storage, small aliquots suitable for single use were stored at −80 °C. 

### 3.4. Cloning, Expression and Purification of (DAF)_2_-Fc (v2) from Nicotiana benthamiana.

The sequence encoding the short consensus repeats 1–4 of human DAF (residues 35–286; GenBank accession number P08174) was cloned upstream and in-frame of the human IgG1 hinge/Fc sequence. Both DAF and Fc sequences were codon-optimized for expression in tobacco. The Fc encoded the mutation N297➔Q (numbering according to Kabat *et al.* [40]), to produce a non‑glycosylated Fc protein. The DAF-Fc sequence was cloned into the pTRAkc plant expression vector [41], and then transformed into *Agrobacterium tumefaciens* GV3101 (pMP90RK) by electroporation. The final construct included sequences encoding a 5' signal peptide and a 3' SEKDEL peptide to facilitate accumulation in the plant’s endoplasmic reticulum. 

**Figure 6:**
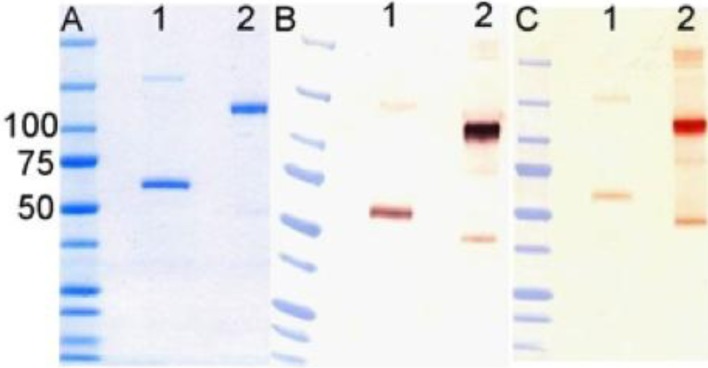
Analysis of purified DAF-Fc. (**A**) 2 µg/lane of (DAF)_2_-Fc (v2) was electrophoresed through SDS-polyacrylamide gel and stained with Coomassie G250. Additional gels with 400 ng (lane 1) or 100 ng (lane 2) were blotted onto nitrocellulose ad probed with (**B**) anti-human IgG or (**C**) anti-DAF antibodies. Lane 1, is protein reduced with DTT; lane 2, non reduced protein. Molecular mass standards are indicated in kDa.

Transient expression of the fusion protein was accomplished by whole-plant vacuum infiltration [42] of *Nicotiana benthamiana* using the transformed *A. tumefaciens* strain carrying DAF-Fc, with co‑infiltration of an *A. tumefaciens* strain carrying the p19 silencing suppressor gene of tomato bushy stunt virus [43] to prolong and amplify expression. After infiltration, the plants were maintained in the greenhouse under standard conditions for 7 days prior to protein purification. *N. benthamiana* leaves were harvested, washed in ice water and blotted dry, then homogenized in a blender with extraction buffer (150 mM NaCl, 50 mM sodium phosphate, 10 mM sodium thiosulphate, 1 mM phenylmethylsulfonyl flouride, pH 7.4). The homogenate was filtered and the filtrate centrifuged at 15,000 g for 30 min at 4 °C. The clarified juice was recovered and pumped over a column of Protein A-Sepharose 4B (Life Technologies, Carlsbad, CA, USA). The column was washed with PBS and eluted with 10 mM glycine, pH 3.0. Purified protein was analyzed using standard methods. Samples were subjected to SDS-polyacrylamide gel electrophoresis (under reducing and non-reducing conditions) and visualized by Coomassie G250 staining, or by western blot analysis using antibodies against human IgG1 and DAF (Figure 6). Purified of (DAF)_2_-Fc was visualized by Coomassie G250 staining, or by western blot analysis using antibodies against human IgG1 and DAF (Figure 6). 10–20 µM samples of (DAF)_2_-Fc −v2 were stored in 20 µL aliquots.

#### 3.5. Fluorescent Labeling of (DAF)_2_-Fc

1.8 µM (DAF)_2_-Fc in 200 µL sodium bicarbonate buffer (pH 8.3) was mixed with 20 µL of 1 mg/mL amine-reactive Alexa Fluor 488 carboxylic acid succinimidyl ester in DMSO for 30 min at room temperature. The fluorescently tagged (DAF)_2_-Fc was purified and concentrated by ultra filtration in phosphate buffered saline using a 30,000 NMWCO Centricon membrane. The fluorophore to protein (f/p) ratio was determined following standard procedures supplied by the manufacturer. The f/p ratio was 2.8:1. The quantum yield (ϕ) of (DAF)_2_-Fc^Alexa488^ was measured relative to fluorescein with absorbance matched samples using a Hitachi model U-3270 spectrophotometer (San Jose, CA, USA) and a Photon Technology International QuantaMaster model QM-4/2005 spectrofluorometer (Lawrenceville, NJ, USA). 

### 3.6. Cell Culture

Tanoue B, and Vero E6 were maintained in minimum essential media (MEM) (GIBCO, Grand Island, NY, USA). All media contain 10% heat-inactivated fetal bovine serum (FBS), 100 units/mL penicillin, 100 mg/mL streptomycin, 10 mM HEPES, pH 7.4, 20 mg/mL ciprofloxacin, 2 mM L-glutamine, at 37 °C in a water jacketed 5% CO_2_ incubator. 

### 3.7. Confocal Microscopy Imaging

Confocal laser scanning microscopy was performed with Zeiss META or LSM 510 systems using 63 × 1.4 oil immersion objectives as previously described [44]. 

### 3.8. Cytometry Experiments

Equilibrium and kinetic binding interactions were analyzed by flow cytometry as described previously [17,45] using BD FACScan and Accuri C6 flow cytometers. Here we provide the essential elements of cytometric analyses of the binding of fluorescent ligands (DAF)_2_-Fc^Alexa488^ and SNV^R18^ to beads and cell surface receptors. A flow cytometer was used to determine the concentration (beads/µL) of Protein G and streptavidin-coated beads in their respective stock suspensions before application. For analyses of equilibrium ligand binding, fluorescence histograms of 500–3000 beads were recorded as a function of ligand concentration at steady state. The flow cytometer’s capacity to discriminate between free and bound ligand without a wash step enables real time addition of ligands to bead suspensions where binding is manifested by exponential increase in mean/median channel fluorescence. The increase in the mean fluorescence channel number of the histogram is related to the fractional receptor occupancy of the specifically bound ligand minus the mean fluorescence channel number of beads exposed to fluorescent ligand under identical conditions but in the presence of excess non-fluorescent ligand (at a concentration of at least 1000 × *K_d_*). To conserve scarce reagents, the use of blocking reagents at 1000 × *K_d_* can be obviated by using beads that lack the cognate receptor, such as our use of streptavidin beads as controls for protein G beads herein.

The number of receptors per bead or cell was calibrated using a variety of calibration beads depending on spectroscopic region of interest. Commercial standards from Bangs Laboratories (Fishers, IN, USA), Quantum fluorescein isothiocyanate (FITC) MESF (molecules of equivalent soluble fluorescein) beads were used with the appropriate correction factor, to account for the spectroscopic differences between calibration standards and probes detected in the 515–545 nm band pass filter-delimited spectral region [36]. Thus, the correction factor (cf) appropriate for Alexa488 was calculated using Equation (3),

*cf =* (*ε_ex_ϕ%T*)*_Alexa488_/*(*ε_ex_ϕ%T*)*_fluorescein_* = (54,750 × 0.99 × 28)/(75,680 × 1 × 28) = 0.71 (3)

where ε_ex_ is the absorption coefficient of the fluorophore at the excitation wavelength, *ϕ* is the quantum yield of the target fluorophore, and %T is the percentage fraction of fluorescence light transmitted by the 530-nm, 30-nm-wide bandpass filter. Using the 488 nm laser excitation, the off-resonance excitation of the Alexa488 was at 75% of its maximum compared to fluorescein, which was excited at 88% of its absorption maximum. The quantum yield of target fluorophores were measured relative to standard solutions such as fluorescein or rhodamine as described for (DAF)_2_-Fc^Alexa^ above. For probes detected in spectral regions other than the 515–545 nm window covered by MESF beads, we used quantum dot calibration beads or lipobeads [27]. As reported elsewhere, the emission quantum yield of quantum dots varies among batches and manufactures, thus the *cf* is batch specific. Lipobeads were custom made, as described below, for calibrating virus binding to beads or cells. In our application lipobeads use the same R18 fluorophore in the same envelope-mimetic lipid environment as the virus particle [17]. Thus the *cf* value is 1. 

### 3.9. Supported Bilayer Membranes on Glass Beads

Lipid coated glass beads (lipobeads) were prepared as previously described [17,27]. For the present study, lipobeads comprising either a single-component DOPC lipid bilayer membrane or a 1:1:1 ternary mixture of DOPC/sphingomyelin/cholesterol (DSC) lipid membranes doped with a variable mole fraction [27] of (e.g., 2.7 mole %) R18 were prepared for use as flow cytometry calibration standards for quantifying virus particles bound to protein G/(DAF)_2_-Fc beads.

### 3.10. Equilibrium Binding of Molecular Assembly Components (DAF)_2_-Fc, and SNV^R18^ on Protein G Beads

*(DAF)_2_-Fc.* To evaluate the equilibrium binding of (DAF)_2_-Fc to protein G, 10,000 beads were added into several 20 µL volume samples, and then mixed with increasing concentrations of (DAF)_2_-Fc^Alexa488^ ranging from ~1 nM–1 µM for an hour at room temperature. Streptavidin-coated beads were used as controls for measuring non-specific binding. After incubation, the samples were diluted to 50 µL and read on the flow cytometer*.* The site occupancy of (DAF)_2_-Fc^Alexa488^ was determined from analyzing cytometry histograms of samples measured against standard calibration beads as described above. 

*SNV^R18^.* The determination of the saturable binding site density of (DAF)_2_-Fc^Alexa488^ on protein G beads facilitated the next step of producing the molecular assembly platform for displaying SNV^R18^ from incubating 760,000 Protein G beads with a 100-fold stoichiometric excess of (DAF)_2_-Fc for 30 min at room temperature. The molecular assembly beads were then washed once and resuspended in HHB buffer at 1000 beads/µL. Subsequently, 10 µL aliquots from this stock were incubated with increasing titers of virus particles until a plateau signifying binding saturation was reached. Competitive binding experiments were performed with a single concentration of bead-borne SNV^R18^ and a variety of concentrations of soluble recombinant DAF in order to generate a competitive binding curve, from which to determine the monovalent affinity of soluble DAF. The dissociation constants for the binding of the competitor were determined by examination of the fractional occupancy of SNV^R18^, assuming a single class of binding sites. 

### 3.11. Cell Surface Distribution of DAF and α_v_β_3_ Integrins

Adherent cells (Vero) were detached with accutase (Sigma), blocked with 10% human serum for 1 h, and incubated with primary antibodies at 4 °C on a rotator for 2 h. Secondary antibodies were incubated at 4 °C on a rotator for 1 h. The cells were washed once and then analyzed with an Accuri C6 flow cytometer. Non-specific binding by secondary antibodies was determined by staining cells with isotype controls in the absence of primary antibodies (see Table 1 notes).

### 3.12. Virus Binding to DAF Expressed on Tanoue B Cells

Binding assays were performed by incubating varying concentrations of SNV^R18^ with 10,000 Tanoue B cells in microfuge tubes gently nutating for 30 min. Specific binding to DAF expressed on Tanoue B cells was blocked with 40 µg/mL of rabbit H319 anti-DAF antibodies [17]. SNV^R18^ particles were also pre-incubated with sDAF and (DAF)_2_-Fc to evaluate their comparative capacities to inhibit binding to cellular DAF. Paired samples of fluorescently labeled SNV^R18^ particles were incubated with titers of sDAF and (DAF)_2_-Fc for one hour and then mixed with 10,000 Tanoue B cells in 50 µL for 30 minutes and then read on a flow cytometer.

### 3.13. Infectivity Assays

For BSL3 live virus infection assays, Vero E6 cells were infected with SNV strain SN77734 inocula (moi = 0.1) that had been preblocked with 0–3.35 µM soluble DAF (sDAF) in a final volume of 100 µL for 1hour. Unbound virus particles were removed by a triple washing, and cells were then transferred to a CO_2_ incubator for 24 h. The infection was monitored by a standard Western blot analysis of N-protein expression where cell lysates were boiled in sodium dodecyl sulfate buffer and separated by 10% sodium dodecyl sulfate-polyacrylamide gel electrophoresis, transferred to a nitrocellulose membrane. SNV N protein was detected with αSNV/N, (hyperimmune rabbit anti-SN virus N protein) [46] used at a 1:1000 dilution, with overnight incubation, washed and then probed for an hour incubation with a secondary antibody (Peroxidase AffiniPure Goat Anti Rabbit IgG; cat# 111-035-003, lot# 104668, used at 1:1000 dilution) from Jackson ImmunoResearch Laboratories (West Grove, PA) The nitrocellulose membrane was then treated with an HRP substrate from Pierce: SuperSignal ® West Pico (Product # 0034077) for 5 min before imaging. Quantitative analysis of the gel was performed using A Biorad Molecular Imager, ChemiDoc XRS+ equipped with *Image Lab Software 4.1* [38].

### 3.14. Ligand Binding Kinetic Measurements

The time course of association was measured by acquiring a 60–180 s baseline of ligand-free beads before an aliquot of (DAF)_2_-Fc^Alexa488^ was added with a Hamilton syringe, while data collection continued for up to 600 s. This process was repeated for different concentrations of (DAF)_2_-Fc^Alexa488^. Raw data were converted to ASCII format, using software developed by Dr Bruce Edwards at UNM [17]. The dissociation rate constant was measured by adding a large excess of soluble protein G to (DAF)_2_-Fc^Alexa488^-bearing Protein G beads, during a data collection time course. The same process was repeated to measure the binding kinetics of SNV^R18^ to (DAF)_2_-Fc bearing beads. 

### 3.15. Kinetic Modeling of Virus Binding

Hantaviruses encode two glycoproteins, Gn and Gc, which are required for receptor binding. We used the structural parameters from recent studies of Tula [36] and Hantaan viruses [37] to estimate site occupancies of Gn-Gc heterodimers on 192 nm—diameter SNV virus particles used in our study [17]. Assuming a suggested upper limit of 70% surface coverage of the particle by Gn-Gc spikes [36] we estimated an average surface expression of 562 Gn-Gc heterodimers on SNV. For the purposes of our present study, this number of unique Gn-Gc sites constitutes the valency (v) of each single virus particle. Upon binding to the surface of a bead, only 2 out of the ~562 Gn-Gc heterodimers can simultaneously engage the bivalent (DAF)_2_-Fc receptor on the same bead, thus the *effective* valency of the bound SNV is limited to 2. 

**Figure 7:**
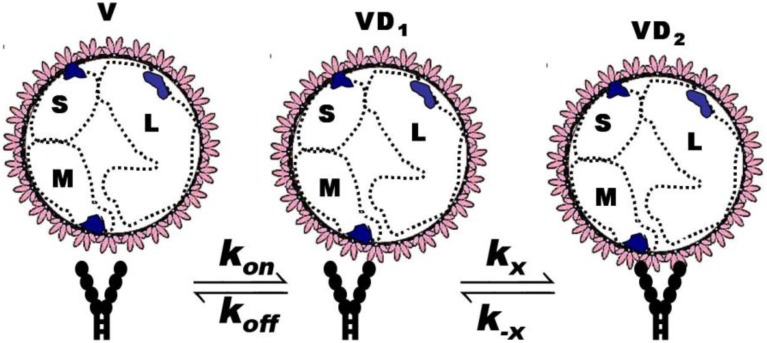
Model binding of multivalent virus (V) to bivalent (DAF)_2_-Fc on beads occurs in two steps (S, M, L refer to *small*, *medium*, and *large* RNA segments of the viral genome) [47]. First, virus particles in solution at concentration [V], each present an estimated 562 Gn-Gc spikes that, with equal probability, can bind to free bead-borne (DAF)_2_-Fc sites with constants, *k_on_* and *k_off_*. Second, once singly bound, the virus-DAF complex [VD_1_] can form [VD_2_] by binding to the second DAF with rate constants *k_x_* and *k_−x_.*

It is also possible to consider the ligation of more than one (DAF)_2_-Fc receptor on the bead. This might increase the valency to 2 + n (where n is the nominal value of additional DAF binding sites). Based on topographical constraints and goodness of fit of a simple model, the multi (DAF)_2_-Fc ligation model was not considered. The present scheme is illustrated in Figure 7 Considering a simple binding model as shown in Equation (4) [48,49,50], the following mass action rate equations were used to determine the kinetic changes to the free and bound concentration of virus particles, [V], and [VD_i_] *i* = 1,2. Here, *k_on_* and *k_off_* are the bimolecular binding and dissociation rate constants of one footed binding, whereas *k_x_* and *k_−x_* are the forward and reverse rate constants describing the cross-linking binding of the second Gn-Gc heterodimer to the free binding site of (DAF)_2_-Fc.

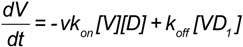
(4a)

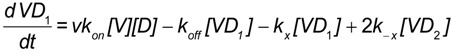
(4b)

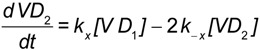
(4c)

Assuming that *k_on_* << *k_x_*, the concentration of VD_1_ is expected to approach steady state values (see Appendix A), which allows the model to be simplified under quasi-steady state regime considerations [30]. In this way the two-footed binding of SNV to (DAF)_2_-Fc was reduced to a two parameter fit, where k_off’_ is the dissociation rate constant of SNV from (DAF)_2_-Fc.

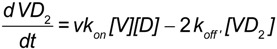
(5)

The experimental rate equations for the variable parameters in Equation (5) were solved numerically using the Runge-Kutta method [51] for values of reaction rate constants *k_on_* and *k_off’_* using *Berkeley Madonna Software* [52]. 

## 4. Conclusions

This study has described a generalizable cell-free platform to characterize specific binding interactions between DAF and SNV. The derived equilibrium and kinetic binding constants from this study provide useful insights into future studies on the mechanism of DAF’s co-receptor role in mediating cell entry. 

## Appendix A

Application of the steady state approximation to the association of SNV with (DAF)_2_-fc beads.

From Equation (4b):

(A1)

(A2)

*k_x_ >> k_off_* and *k_x_ >> k_-x_* Equation (A2) simplifies to:

(A3)

and substituting (A3) into Equation (2c) yields

(A4)
