# Correction: Buranda, T. *et al*. Equilibrium and Kinetics of Sin Nombre Hantavirus Binding at DAF/CD55 Functionalized Bead Surfaces. *Viruses* 2014, *6*, 1091-1111

**DOI:** 10.3390/v6062463

**Published:** 2014-06-20

**Authors:** Tione Buranda, Scarlett Swanson, Virginie Bondu, Leah Schaefer, James Maclean, Zhenzhen Mo, Keith Wycoff, Archana Belle, Brian Hjelle

**Affiliations:** 1 Department of Pathology, University of New Mexico School of Medicine, MSC08 4640, Albuquerque, NM 87131, USA; E-Mails: scswanson@salud.unm.edu (S.S.); vbondu@salud.unm.edu (V.B.); bhjelle@salud.unm.edu (B.H.); 2 Cancer Center, University of New Mexico School of Medicine, MSC08 4640, Albuquerque, NM 87131, USA; 3 Center for Infectious Diseases and Immunity, University of New Mexico School of Medicine, Albuquerque, NM 87131, USA; 4 Planet Biotechnology Inc., 25571 Clawiter Road, Hayward, CA 94545, USA; E-Mails: leah.Schaefer@gmail.com or lschaefer@planetbiotechnology.com (L.S.); jmaclean@planetbiotechnology.com (J.M.); zmo@planetbiotechnology.com (Z.M.); kwycoff@planetbiotechnology.com (K.W.); abelle@planetbiotechnology.com (A.B.)

The procedure for blocking infection with H319 antibodies in polarized Vero E6 cells, grown in transwell inserts, was erroneously omitted on page 1104, Section 3.13. (entitled ‘Infectivity Assays’) of [1]. It is important to note that robust blocking with H319 anti-DAF anti-bodies, as shown in Figure 5B, was measured in polarized cells, seeded on filter supports in transwell plates, as described below.

For *effective* blocking of hantavirus infection with H319 antibodies, Vero E6 cells were seeded (1.5 × 10^5^ cells/well) in 6-mm polyester 0.4 micron transwell inserts (Corning Life Sciences, Tewksbury, MA) and allowed to grow to confluence and polarize for 3–4 days. Formation of tight junctions was evaluated by measuring an increase in transmonolayer electrical resistance (TER) across the cell monolayer, using a voltohmmeter coupled to an Endohm sensor chamber (World Precision Instruments Inc., Sarasota, FL, USA) [2]. Cells were then infected by adding, SNV or HTN inocula (moi = 0.1) to the apical chamber of the transwell plate. To block binding to DAF expressed on the apical cell surface, cells were pretreated with 2.6 µM H319 anti-DAF antibodies for an hour at 37 °C before adding virus.

The figure caption in Figure 5B should now read: Western blotting of *polarized*, Vero E6 cells infected with HTN and SNV (control), and cells pretreated with DAF blocking antibody (2.6 µM H319).
